# Should household air quality monitoring be considered in selected patients with asthma and COPD?

**DOI:** 10.1136/bmjresp-2025-003899

**Published:** 2026-07-01

**Authors:** Rubèn González-Colom, Alba Gómez-López, Alicia Aguado, Néstor Soler, Núria Sánchez-Ruano, Marta Sorribes, Antonio Montilla-Ibarra, María Figols, Alberto Rodríguez, Emili Vela, Jordi Piera-Jiménez, Ramón Farre, Josep Roca, Isaac Cano, Jose Fermoso, Ebymar Arismendi

**Affiliations:** 1 Fundació de Recerca Clínic Barcelona - Institut d’Investigacions Biomèdiques August Pi i Sunyer (FRCB-IDIBAPS), Barcelona, Spain; 2 University of Barcelona, Barcelona, Spain; 3 CARTIF Technology Center, Valladolid, Spain; 4 Pulmonology Department, Hospital Clinic de Barcelona, Barcelona, Spain; 5 Consorci d’Atenció Primaria de Barcelona-Esquerra (CAPSBE), Barcelona, Spain; 6 CAP Numancia, Institut Catala de la Salut (ICS), Barcelona, Spain; 7 inBiot, Pamplona, Spain; 8 Centro de Investigaciones Energéticas Medioambientales y Tecnológicas (CIEMAT), Madrid, Spain; 9 Catalan Health Service, Barcelona, Spain; 10 Digitalization for the Sustainability of the Healthcare System (DS3), Barcelona, Spain; 11 Centro de Investigación Biomédica en Red de Enfermedades Respiratorias (CIBERES), Instituto de Salud Carlos III, Madrid, Spain

**Keywords:** Asthma, COPD

## Abstract

**Background:**

Indoor air quality (IAQ) is a well demonstrated actionable determinant of health status. Low-cost sensors (LCS) could enable patient-centred assessments, but real-world clinical utility is uncertain.

**Objective:**

Evaluate the feasibility, usability and clinical indications of home IAQ monitoring with LCS.

**Methods:**

We conducted a cohort study involving household continuous IAQ monitoring with LCS of 205 adults with chronic obstructive pulmonary disease, bronchiectasis or asthma. Household IAQ was profiled prospectively over a 2-month period registering concentrations of carbon dioxide, particulate matter 2.5 µm (PM_2.5_) and formaldehyde every 10 min. For each pollutant, dwellings were classified as good, moderate or unhealthy according to the Global Open Air Quality Standards thresholds. Pulmonary exacerbations requiring unplanned hospitalisations and all-cause emergency department (ED) visits over the preceding 12 months were registered and potential relationships with household IAQ results were explored.

**Results:**

Of the 205 participants, 178 were included in the final analysis after excluding dropouts and cases with insufficient monitoring data. More than half of homes (51.7%) had at least one pollutant in an at-risk category. The burden was mostly generated by PM_2.5_: 40.1% of dwellings were classified as at risk (32.8% moderate; 7.3% unhealthy). Formaldehyde exceeded the low-risk threshold in 22 homes (12.4%). Tobacco smoking, either active or passive, was significantly associated with PM_2.5_ levels (p<0.001). No relationships were found between IAQ categories and hospitalisations nor with all-cause ED visits.

**Conclusions:**

LCS are useful tools for short-term, targeted household IAQ screening in chronic respiratory patients. Indoor pollution is highly prevalent and largely PM_2.5_ driven. Further research is needed to assess the short-term health impacts of these exposures.

**Trial registration number:**

NCT06421402.

**WHAT IS ALREADY KNOWN ON THIS TOPIC**

- Indoor air pollution constitutes a major environmental determinant of respiratory morbidity, with evidence indicating that reducing exposure through improved indoor air quality (IAQ) can attenuate associated health risks. The potential of low-cost sensors (LCS) for identifying modifiable exposures in patients with chronic respiratory disease has been suggested, but their real-world feasibility and clinical value to inform preventive care interventions are still uncertain.

**WHAT THIS STUDY ADDS**

- This study supports the feasibility and operational reliability of LCS for short-term household IAQ screening in chronic respiratory patients. Whereas, indoor pollution was found to be highly prevalent, mainly driven by fine particulate matter and indoor smoking, suggesting potential value for targeted exposure mitigation actions.

**HOW THIS STUDY MIGHT AFFECT RESEARCH, PRACTICE OR POLICY**

- These findings highlight the relevance of IAQ as a potentially modifiable component of chronic respiratory care and support further evaluation of environmental assessments within preventive management strategies. Further research should explore short-term health impacts and develop actionable guidelines for integrating IAQ monitoring into clinical and public health strategies.

## Introduction

 Exposure to air pollution is considered the most important environmental health risk factor with significant deleterious impacts on physiological developments during early life and accelerated functional decline in the elderly.^1^ There is also robust evidence of both acute and sustained adverse effects of air pollution on several organs and systems, with respiratory disorders being one of the primary concerns.^2^,^6^ Specifically, the effects of exposure to particulate matter (PM), as well as oxidants like nitrogen oxides (NOx) and ozone (O_3_), have been extensively demonstrated.^7^ The exposure to formaldehyde (CH_2_O) is also associated with asthma diagnosis and exacerbations.^8 9^ Furthermore, there is growing attention on the negative impact of other airborne pollutants, such as volatile organic compounds (VOCs).^10^

Due to the magnitude of the burden of air pollution on human health, international agencies^11^,^14^ are actively deploying public health policy actions. In this context, research on indoor air quality (IAQ) is raising interest due to unknowns on the sources of indoor pollution,^15 16^ the interactions between IAQ and outdoor air quality (OAQ), and most importantly, the lack of available information to generate appropriate regulations and health policies on IAQ, as reported in two recent official statements of the American Thoracic Society.^17 18^ Likewise, the European Union launched the IDEAL Cluster in 2022,^19^ an ambitious research and innovation programme encompassing seven consortia working on coordinated action plans to set IAQ standards. One of the relevant areas of action of the IDEAL cluster is to explore the potential of low-cost sensors (LCS) for IAQ monitoring in health-related applications. LCS are gaining traction due to their potential for remote continuous IAQ monitoring of different pollutants, affordability, applicability and potential for widespread deployment in different scenarios.^20^,^22^ The harmful effects of IAQ on respiratory health have been consistently proven in patients with chronic obstructive pulmonary disease (COPD)^523^,^25^ showing significant associations between indoor pollutants and symptoms, functional capacity and risk of exacerbations.

Interestingly, the CLEAN AIR^23^ study showed potential health benefits of portable particulate air cleaners. A recent report highlighted that the harmful effects of PM pollution on cardiovascular health in patients with COPD can be mitigated by reducing exposure,^4^ suggesting a potential role for household IAQ monitoring in high-risk patients. As stated, LCS might open a window of opportunity to enhance the management of selected patients with chronic obstructive respiratory diseases. It is acknowledged, however, that there are uncertainties regarding the quality of LCS measurements, and their potential for applicability in healthcare.^20^,^22^ Accordingly, our primary objective was to evaluate the feasibility and usability of home IAQ monitoring with LCS, as well as to explore its potential relevance for clinical management.^26^,^28^

## Method

### Study cohort

Between October 2023 and March 2025, we enrolled 205 patients with COPD or bronchiectasis or asthma from two complementary settings. Most participants (n=152; 77%) were recruited through four primary care centres in the Barcelona-Esquerra Integrated Care Area,^29^ each serving approximately 20 000 residents. These patients were identified in the Catalan Health Surveillance System (CHSS)^30^ with a diagnosis International Classification of Diseases, Tenth Revision, Clinical Modification (ICD-10-CM),^31^ J44 (COPD), J45 (asthma) and J47 (bronchiectasis) and classified as high-risk due to disease severity and/or multimorbidity, defined as an Adjusted Morbidity Groups (AMG)^32^,^34^ score at or above the 80th percentile of the regional risk pyramid. The remaining patients (n=53; 23%), all with stage 5 or 6 asthma,^35 36^ were enrolled at the Severe Asthma Unit at Hospital Clinic de Barcelona.

Eligibility criteria, recruitment procedures and monitoring protocols are detailed in the published study protocol.^28^ Throughout the study, clinical management followed international recommendations for COPD,^37 38^ bronchiectasis,^39^ asthma^35 36^ management.

### Study design and IAQ evaluation

**Figure 1:**
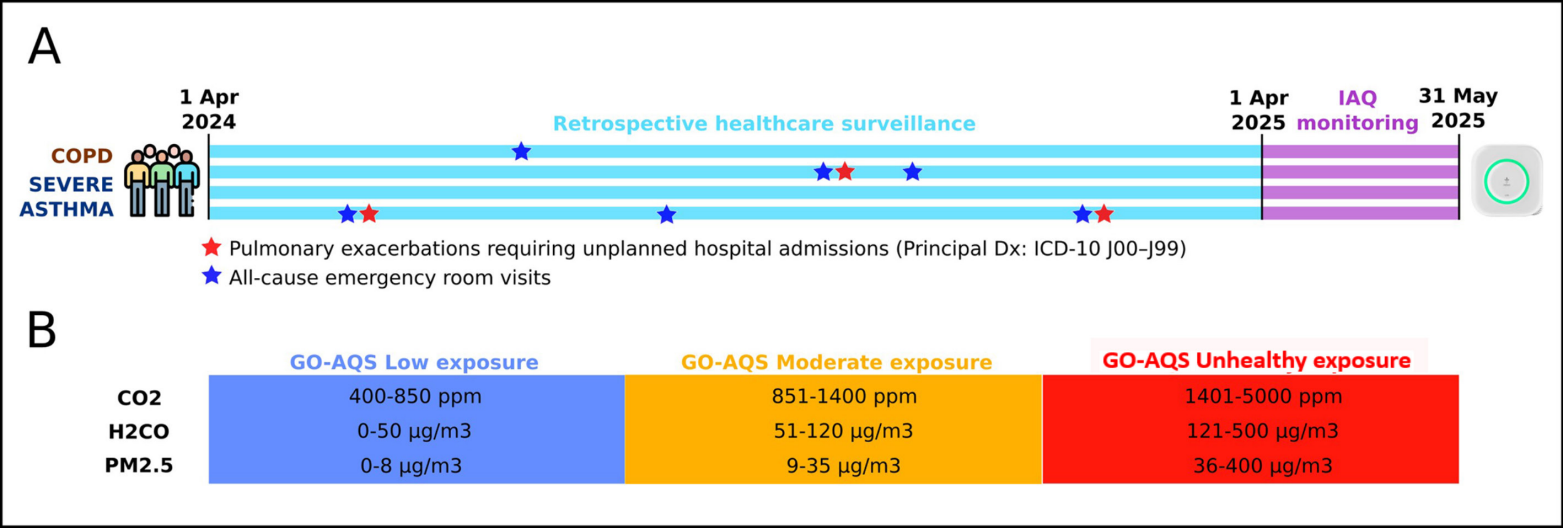
Study timeline and design. (**A**) Prospective in-home IAQ monitoring (purple) was conducted from 1 April to 31 May 2025. Retrospective surveillance of acute respiratory events (light blue; 1 April 2024–31 March 2025): severe pulmonary exacerbations leading to unplanned hospital admissions (ICD-10-CM J00–J99; red stars) and all-cause emergency department visits (blue stars). (**B**) Long-term exposure thresholds from the Global Open Air Quality Standards (GO-AQS) are used to classify household IAQ as Good, Moderate or Unhealthy for CO₂, formaldehyde and PM_2.5_. CO₂, carbon dioxide; COPD, chronic obstructive pulmonary disease; IAQ, indoor air quality; ICD-10, International Classification of Diseases, Tenth Revision; PM_2.5_, particulate matter 2.5 µm.

The study was conducted and reported in accordance with Strengthening the Reporting of Observational Studies in Epidemiology (STROBE)^40^ guidelines. The study design consisted of the prospective characterisation of household IAQ in the study cohort using continuous LCS measurements during a 2-month period. The relationships between dwelling IAQ levels and unplanned hospitalisations due to respiratory exacerbations, as well as all-cause emergency department visits occurred during the previous twelve months, were explored (figure 1).

#### Prospective in-home IAQ monitoring (1 April 2025–31 May 2025)

Between 1 April and 31 May 2025, prospective in-home IAQ monitoring was conducted in patient dwellings using MICA-IBIOT’s^41^ LCS to continuously record key IAQ parameters every 10 min, including temperature, relative humidity, carbon dioxide (CO₂), PM_1_, PM_2.5_, PM_10_, formaldehyde and total VOCs concentrations. Online supplemental material provides detailed information on the manufacturer specifications of the IAQ sensors used and describes the validation work conducted within the K-HEALTHinAIR project,^42^ comprising chamber tests and in-field comparisons, to assess accuracy, linearity, inter-sensor agreement and drift. In short, the sensor performance for PM and formaldehyde met predefined quality criteria and was adequate for the study objectives; by contrast, total VOC measurements failed quality thresholds and were excluded from analysis.

To assess indoor pollution levels and stratify homes according to their IAQ quality for different parameters, we applied the reference framework outlined in the Global Open Air Quality Standards (GO-AQS) white paper.^43^ The GO-AQS framework was selected for its operational suitability in continuous monitoring contexts, enabling improved stratification of exposure severity through multilevel classification and facilitating the integration of multi-pollutant profiles, while remaining broadly consistent with WHO guideline ranges.

Subsequently, dwellings were classified using the GO-AQS framework adapted for long-term exposure assessment of the key pollutants monitored: CO₂, formaldehyde and PM_2.5_. The concentration of each pollutant in the patient’s home was classified in three categories: (1) good, (2) moderate or (3) unhealthy ranges. Homes were classified based on their all-time average pollutant concentrations and stratified according to their IAQ status for each parameter. Additionally, the relative frequency of time spent in each risk category was calculated to provide a more detailed analysis of exposure patterns and variability.^44 45^ For IAQ analyses, we excluded the cases with less than 20 complete monitoring days and those patients who discontinued follow-up.

#### Retrospective clinical surveillance (1 April 2024–31 March 2025)

To examine the associations between household indoor pollution and severe health events, we retrieved from the CHSS the registries of acute care events over the preceding year (1 April 2024–31 March 2025). The primary outcome was severe pulmonary exacerbations requiring unplanned hospital admission with a principal respiratory diagnosis (ICD-10-CM J00–J99). Planned admissions and non-respiratory urgent admissions were excluded. All-cause emergency department visits were captured as an exploratory outcome.

The analyses assessing the relationship between IAQ and clinical outcomes were exploratory and hypothesis-generating.

### Statistical analysis

Numeric variables are described as mean and SD or median and IQR (IQ1–Q3), depending on their distribution. Categorical variables are summarised as counts and percentages; n (%). The comparison of numerical outcomes between patients with respiratory disease exacerbations requiring hospital admission and those who did not was conducted using Student’s t-test for normally distributed variables or the Mann-Whitney U test for non-normally distributed variables. The frequency distributions of categorical factors were compared using Fisher’s exact test. A p<0.05 was considered statistically significant. All statistical analyses were performed using R, V.4.1.1.^46^

## Results

### Characteristics of the study cohort

During the prospective IAQ monitoring window reported in this manuscript, 27 participants out of the 205 patients included in the cohort (13.2%) were not evaluable, yielding a final analytic sample of 178 patients (86.8%). Of these, 130 (73.0%) were from the community programme and 48 (27.0%) from the severe asthma unit. The exclusion reasons are the following: 19 (9.3%) dropped out, 3 (1.5%) died and 5 (2.4%) were excluded because of IAQ sensor malfunction or insufficient data.

Table 1 summarises baseline characteristics of the study cohort (n=178), stratified by patients with one or more unplanned hospitalisation due to a severe pulmonary exacerbation (n=50; 28.1%) versus those without such events (n=128; 71.9%). Over the 1-year retrospective surveillance, 69/178 (38.8%) patients accounted for 113 hospitalisations: 31/113 (27.4%) were planned and 82/113 (72.6%) were urgent. Among urgent admissions, 68/82 (82.9%) were due to respiratory exacerbations, corresponding to 50/178 (28.1%) patients; the remaining 14/82 (17.1%) urgent admissions were for non-respiratory causes.

**Table 1:** Main features of the study group: comparisons between patients showing unplanned hospitalisations during the study period (n=50) and all the other patients (n=128)

Variable	All other patients (n=128)	Patients with unplanned hospitalisations (n=50)	P value
Age, gender and smoking habit			
Age; mean (SD)	66.96 (12.17)	68.04 (12.01)	0.596
Male; n (%)	50 (39.06)	25 (50)	0.237
Female; n (%)	78 (60.94)	25 (50)	
Current smokers; n (%)	16 (12.5)	9 (18)	0.304
Former smokers; n (%)	73 (57.03)	31 (62)	
Never smokers; n (%)	39 (30.47)	10 (20)	
Main respiratory diagnosis, comorbidities and forced spirometry			
COPD; n (%)	60 (46.88)	33 (66)	0.166
Asthma, n (%)	11 (8.59)	3 (6)	
Bronchiectasis, n (%)	19 (14.84)	4 (8)	
Severe asthma; n (%)	38 (29.69)	10 (20)	
AMG score; mean (SD)	71.09 (42.48)	97.93 (50.96)	**<0.001**
AMG risk group–very high risk; n (%)	9 (7.03)	11 (22)	**0.007**
AMG risk group–high risk; n (%)	37 (28.91)	19 (38)	
AMG risk group–moderate risk; n (%)	54 (42.19)	15 (30)	
AMG risk group–low risk; n (%)	25 (19.53)	3 (6)	
AMG risk group–very low risk; n (%)	3 (2.34)	1 (2)	
FVC; z-score, mean (SD)	−1.09 (1.28)	−1.46 (1.34)	0.121
FEV1; z-score, mean (SD)	−1.85 (1.37)	−2.51 (1.42)	**0.009**
FEV1/FVC; mean (SD)	62.63 (13.94)	53.28 (17.16)	**<0.001**
Utilisation of healthcare resources during the 12 months before retrospective clinical surveillance			
All-cause hospitalisations; n (%)	32 (25.00)	31 (62.00)	**<0.001**
Unplanned hospitalisations; n (%)	18 (14.06)	29 (58.00)	**<0.001**
Total healthcare expenditure per patient in €; median (Q1–Q3)	4581 (2455–9727)	8541 (5157–14 057)	**<0.001**

Total healthcare expenditure corresponds to total expenditure per year across all healthcare tiers. Bold values indicate statistical significance (p<0.05).

AMG score, Adjusted Morbidity Groups; COPD, chronic obstructive pulmonary disease; FEV1, forced expiratory volume in 1 s; FVC, forced vital capacity.

As displayed in table 1, the patients who experienced at least one unplanned respiratory hospitalisation due to a pulmonary exacerbation along the previous year before the IAQ assessment (n=50) had a substantially higher multimorbidity burden (AMG scoring) and more severe airflow limitation than those without events (n=128). The mean AMG score was higher in the hospitalised group (p<0.001), with an over-representation in the very high risk band (p=0.007). Prior healthcare use in the preceding year was markedly higher among patients with subsequent unplanned admissions: All-cause hospitalisations (p<0.001), unplanned hospitalisations (p<0.001), and greater total healthcare expenditure (p<0.001). Spirometry showed worse obstruction among hospitalised patients: lower forced expiratory volume in 1 s (FEV₁) z-score (p=0.009) and lower FEV₁/forced vital capacity (FVC) (p<0.001), while FVC z-score differences were not significant. Age, sex distribution and smoking status were similar across groups. The distribution of primary respiratory diagnoses was broadly comparable. Collectively, severe pulmonary exacerbations manifested in individuals with greater clinical complexity, expressed as comorbidity burden and previous usage of healthcare resources, and worse airflow obstruction rather than differences in age, sex, smoking habits or diagnostic labels.

### Household IAQ monitoring

#### Monitoring in practice: continuity and technical issues

Among the 183 active participants, 182 were successfully monitored over a 61-day period. However, four additional patients recorded fewer than 20 valid monitoring days and were therefore excluded from the analysis, resulting in the final analytic cohort of 178 participants (table 1). Discontinuities in data monitoring (online supplemental figure S6) were mainly attributed to temporary device disconnections from the power supply, unstable Wi-Fi connectivity and occasional sensor malfunctions or damage. Some incidents resolved spontaneously, while others required patient interaction following telephone assistance or on-site home visits. In a few cases, sensor replacement was needed to restore data transmission. On average, participants missed 5.0 (10.6) monitoring days, corresponding to 8.2% of all possible recording days.

#### Household air quality results and exposure patterns

Table 2 summarises the GO-AQS long-term thresholds for CO₂, formaldehyde and PM_2.5_, defining low, moderate and unhealthy exposure categories. It further reports, for each pollutant, the distribution of homes by category based on dwelling-level mean concentrations, and among these, the number of homes with any smoker present, and finally the overall monitoring time spent in each risk band. Among the 178 monitored homes, 92 (51.7%) exhibited at-risk pollution levels (moderate or unhealthy) for at least one monitored contaminant highlighting the prevalence of poor IAQ. CO₂ levels were generally within safe limits, with 163 (91.6%) homes classified as low risk and 14 (7.9%) falling into the moderate category. Only 1 (0.5%) home exceeded the unhealthy threshold for CO₂. In contrast, formaldehyde pollution was more widespread, with 22 homes (12.4%) exceeding the low-risk threshold, 21 in the moderate category (11.9%) and 1 (0.5%) classified as unhealthy. PM_2.5_ exposure was the most concerning, as 71 homes (40.1%) showed at-risk levels, with 58 in the moderate category (32.8%) and 13 in the unhealthy category (7.3%).

**Table 2:** Results of household indoor air quality monitoring grouped by each of the three risk categories defined by the Global Open Air Quality Standards (GO AQS)^40^

Parameter	Low risk	Moderate risk	Unhealthy risk
CO_2_			
Recommended Thresholds, ppm	400-850	851–1400	1401–5000
Homes in category; n (%)	163 (91.6)	14 (7.9)	1 (0.5)
Homes with any smoker; n (%)	45 (27.6)	2 (14.3)	0 (0)
Time spent in each exposure category, hours; n (%)	200 929 (84.8)	29 955 (12.7)	5870 (2.5)
PM_2.5_			
Recommended Thresholds, μg/m^3^	0–8	9–35	36–400
Homes in category; n (%)	106 (59.9)	58 (32.8)	13 (7.3)
Homes with any smoker; n (%)	10 (9.4)	26 (44.8)	11 (84.6)
Time spent in each exposure category, hours; n (%)	180 734 (76.6)	36 699 (15.6)	18 499 (7.8)
Formaldehyde			
Recommended thresholds, μg/m^3^	0–50	51–120	121–500
Homes in category; n (%)	155 (87.6)	21 (11.9)	1 (0.5)
Homes with any smoker; n (%)	45 (29.0)	2 (9.5)	0 (0)
Time spent in each exposure category, hours; n (%)	196 862 (83.4)	37 410 (15.9)	1665 (0.7)
GO AQS recommendations			
	Ideal air quality–enjoy activities	Reduce sources of pollution. Cut back or reschedule strenuous activities indoors. Ventilate if possible.	Avoid all physical activities (wear N95/FFP3 masks and use personal or central air filtration systems in case of particle pollution or high CO_2_ levels)

CO₂, carbon dioxide; PM_2.5_, particulate matter 2.5 µm.

As depicted in table 2, smokers in dwellings varied across the different air quality risk categories, showing a clear association with PM_2.5_ pollution levels, whereas smoker prevalence showed no increasing gradient across CO₂ or formaldehyde categories. In homes classified as low risk for PM_2.5_, only 10 (9.4%) had smokers, whereas this proportion increased to 26 (44.8%) in moderate-risk homes and reached 11 (84.6%) in unhealthy environments. This strong association (p<0.001) suggests that smoking is a major contributor to fine particulate pollution indoors.

**Figure 2:**
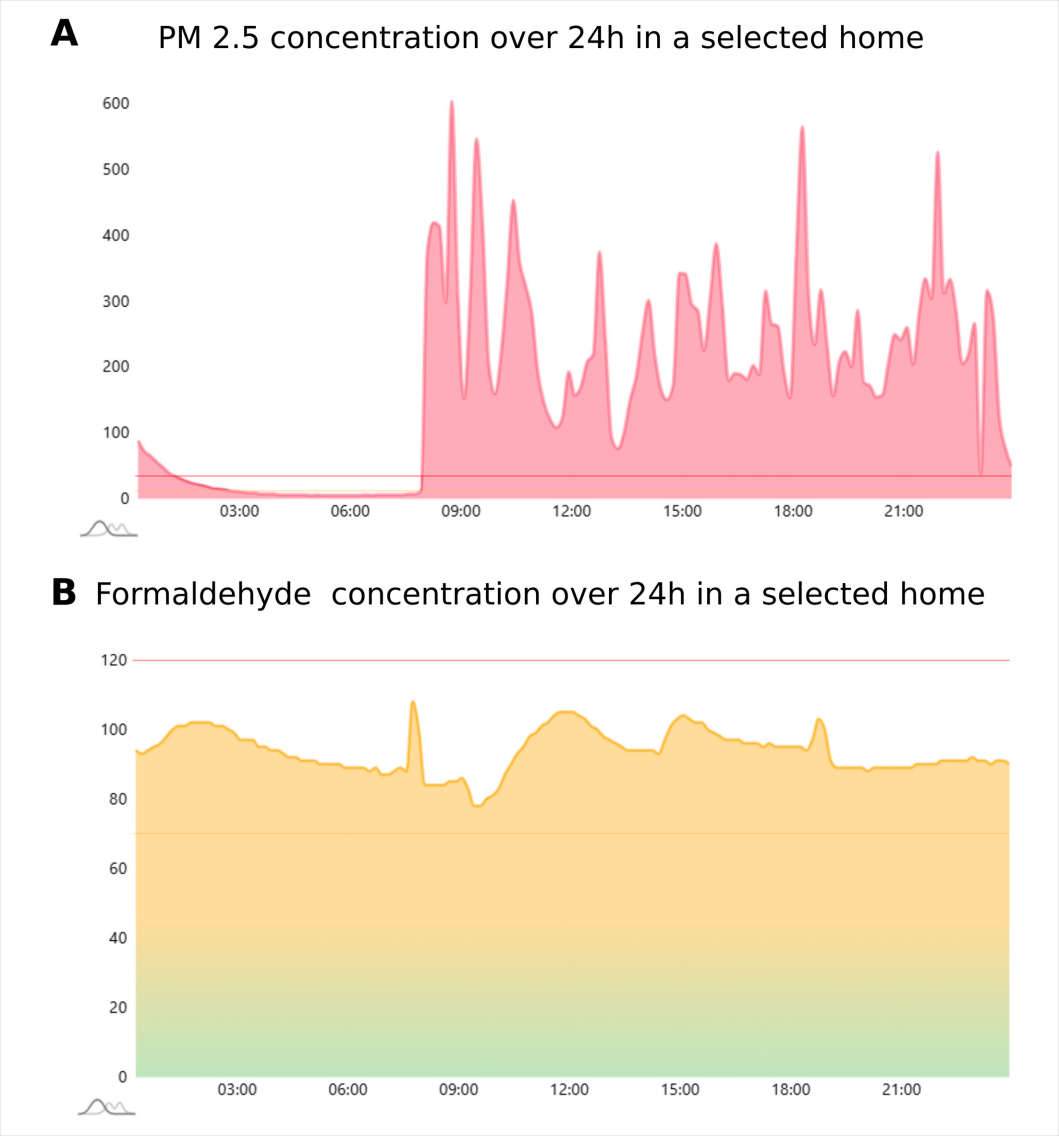
24-hour indoor concentration profiles in selected at-risk homes (μg/m³). (**A**) PM_2.5_ exposure. (**B**) Formaldehyde exposure. The exposure was recorded at 10-minute intervals with MICA-INBIOT LCS; screenshots were exported from the myInbiot monitoring platform. LCS, low-cost sensors; PM_2.5_, particulate matter 2.5 µm.

Analysing the time spent in each risk category suggests distinct emission patterns. PM_2.5_ exposure showed a different pattern, with 59.9% of homes classified as low risk, but spending 76.6% of the time in this category (table 2). This suggests acute pollution peaks driving PM_2.5_ exposure. Figure 2A shows a representative 24-hour PM_2.5_ time series from an at-risk dwelling, illustrating typical indoor exposure dynamics. PM_2.5_ pollution is manifested as pronounced diurnal peaks, exceeding ‘moderate’ or ‘unhealthy’ thresholds for short periods, potentially coinciding with activities such as cooking, cleaning (particularly sweeping or dusting, which resuspends particles), and, most prominently, tobacco use indoors. Other episodic sources included the burning of incense or candles. Night-time concentrations tended to decrease sharply, consistent with particle sedimentation in the absence of human activity.

Figure 2B presents a 24-hour monitoring segment from a dwelling representative of high formaldehyde contamination. Conversely, formaldehyde exposure appears to be continuous, with 87.6% of homes classified as low risk, while spending only 83.4% of the monitored time in this category (table 2). This behaviour reflects passive, continuous emissions, off-gassing, from materials such as furniture, construction products and household goods, rather than direct links to occupant behaviours. These emissions were not associated with short-term activity peaks but could be influenced by environmental conditions such as temperature and ventilation.

**Figure 3:**
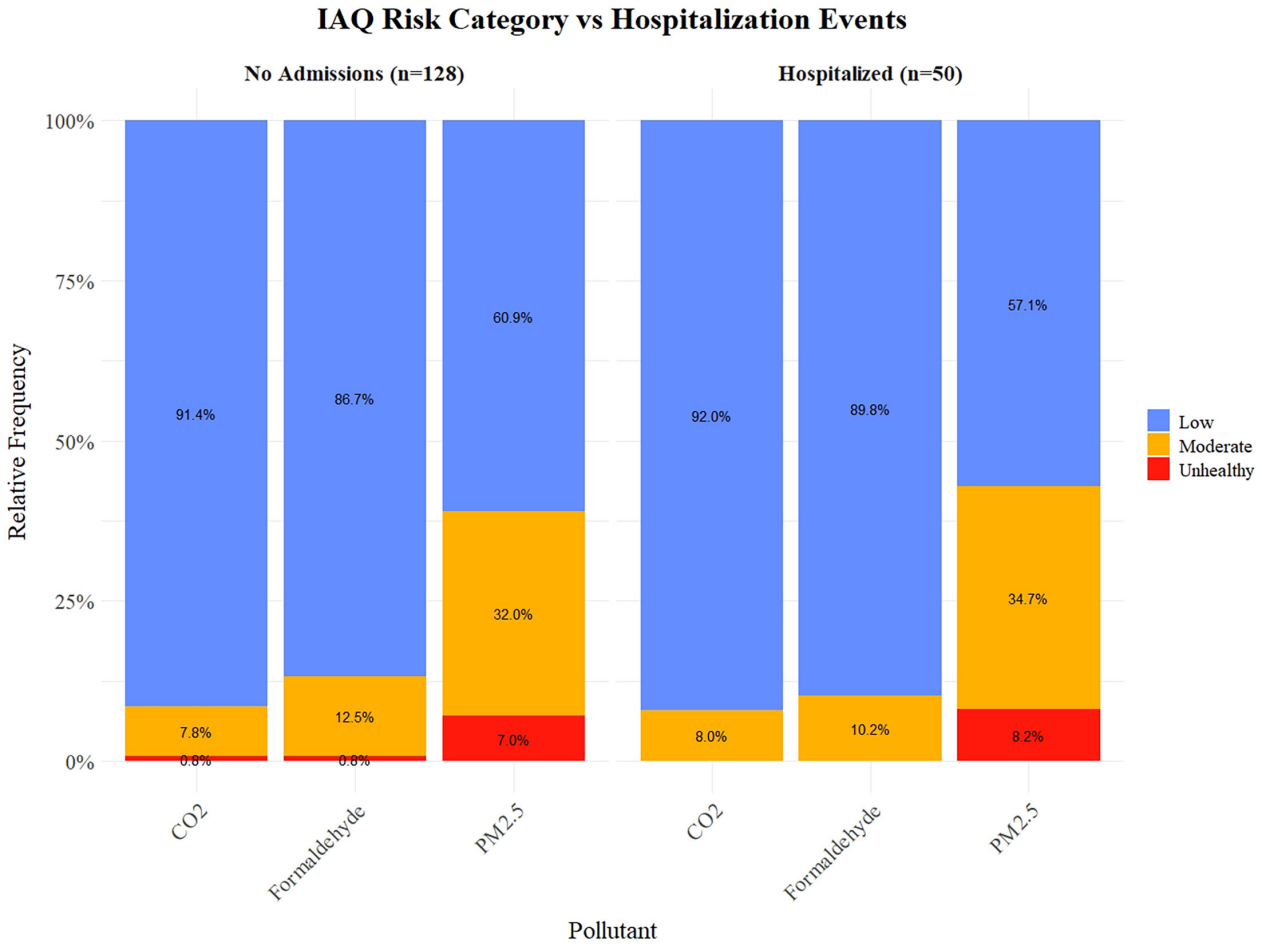
IAQ risk categories by pollutant and hospitalisation status. Stacked bars show the proportion of dwellings in low, moderate and unhealthy GO-AQS categories for each pollutant (CO₂, formaldehyde, PM_2.5_), stratified by patients with no admissions (n=128) versus those hospitalised for severe pulmonary exacerbations (n=50). Percentages within bars indicate the share of homes per risk category in each subgroup. CO₂, carbon dioxide; GO-AQS, Global Open Air Quality Standards; IAQ, indoor air quality; PM_2.5_, particulate matter 2.5 µm.

As shown in figure 3, the distributions of IAQ risk categories for CO₂, PM_2.5_ and formaldehyde were similar in patients who experienced severe pulmonary exacerbations requiring hospitalisation during the previous year of IAQ assessment and those without events. Only PM_2.5_ showed a modest, non-statistically significant, shift toward higher PM_2.5_, exposure among hospitalised patients. Similar distributions were observed when stratifying by all-cause emergency department visits.

Additional multivariable analyses adjusting for age, sex, smoking status, disease severity (FEV₁) and multimorbidity (AMG) yielded results consistent with the univariate findings, with no significant associations observed.

## Discussion

This study reports the operational evaluation of LCS for home-based IAQ assessment and explores the potential clinical relevance of this type of sensor in patients with chronic respiratory disease. This research contributed specifically to two areas: (1) feasibility and readiness of LCS-supported household IAQ monitoring and (2) the role of IAQ in preventive and personalised care.

### Operational feasibility and readiness of LCS

#### Technological readiness

In field deployment, the LCS units provided stable, approximately linear responses within the relevant concentration ranges for PM and formaldehyde, and consistently captured relative changes and temporal patterns, enabling reliable characterisation of household exposure profiles. These observations are consistent with independent evaluations of LCS performance.^20^,^22^ In contrast, total VOC readings tended to overestimate concentrations and showed cross-interference, limiting their suitability for absolute quantification. Nevertheless, they retained qualitative utility in identifying high-emission events (see online supplemental material for details on laboratory assessment of the total VOC sensor). CO₂ levels, on the other hand, primarily reflected indoor occupancy and ventilation status rather than pollution per se, and are best interpreted as indicators of air stagnation or insufficient ventilation within the home.

#### Usability considerations

Some participants experienced temporary data losses due to device disconnections, Wi-Fi instability, sensor malfunction or prolonged absences from home during holiday periods. While most incidents were minor and easily resolved, either automatically, through patient support or by on-site visits, these interruptions highlight the operational demands of maintaining continuous long-term monitoring. Therefore, from an operational perspective, continuous long-term household IAQ monitoring is not cost-effective. Considering the maintenance and synchronisation requirements of IAQ monitors, together with the relative stability of exposure patterns once pollution sources are identified, indefinite monitoring offers limited additional value. We therefore recommend time-limited IAQ assessments, either as an initial screening for patients with difficult-to-control COPD or asthma or as a follow-up to evaluate the impact of targeted environmental interventions.

#### Future improvements

Clinical decision-making would be strengthened by multi-pollutant sensing that includes oxidants (NOx, O_3_). Oxidant gases are associated with respiratory morbidity and can amplify PM-related oxidative stress and airway inflammation when present together, potentially increasing the risk of exacerbations^47^; a multipollutant view is therefore biologically and clinically coherent. Incorporating validated NO₂/O₃ sensing could improve the detection of hazardous indoor conditions and may, in future studies, help inform strategies aimed at preventing acute exacerbations. Equally important is the refinement of VOC sensing. Current VOC sensors are qualitative and non-specific, responding to a wide range of compounds with overlapping signal patterns that make it difficult to identify individual sources or quantify absolute concentrations. This lack of specificity limits their interpretability for clinical or epidemiological purposes.

### The role of IAQ in preventive and personalised care

#### Prevalence and composition of household air pollution

Impaired household IAQ is highly prevalent. More than half of the monitored dwellings exhibited average concentrations above recommended thresholds for at least one of the monitored pollutants. Fine PM was the dominant contributor to poor IAQ, and we identified indoor smoking as a key driver of particulate pollution. The proportion of homes with smokers increased markedly across PM_2.5_ risk categories, from low risk to unhealthy environments, indicating that tobacco smoke is a major driver of indoor particulate pollution. From a clinical and public health perspective, these findings have direct translational implications. Unlike other sources of indoor pollution, tobacco-related exposure can be effectively targeted through well-established interventions. Therefore, integrating IAQ monitoring with smoking cessation strategies and household-level behavioural counselling may represent a feasible and impactful approach to reduce particulate exposure in high-risk respiratory patients. Nevertheless, elevated PM_2.5_ in non-smoker dwellings indicates additional relevant sources, most notably cooking emissions, combustion appliances and cleaning/resuspension, underscoring the need for context-specific mitigation. These results emphasise the relevance of PM exposure beyond tobacco smoke and support tailored recommendations to reduce particulate burden that primarily rely on behavioural change, as most relevant sources are linked to everyday human activities. In contrast, formaldehyde contamination followed a distinct pattern characterised by persistent, thermally dependent off gassing from structural and furnishing materials. Because such emissions are continuous and largely independent of occupant behaviour, mitigation should prioritise source control using low-emission materials, sustained ventilation and temperature regulation to limit volatilisation. In severe cases, gas-phase removal is feasible with sorbent media (eg, activated carbon/chemisorption cartridges).

#### Environmental and clinical determinants of respiratory exacerbations

Hospitalisation risk among complex chronic respiratory patients is primarily determined by the cumulative burden of multimorbidity, the severity of pulmonary impairment and the individual history of exacerbations,^48 49^ and environmental exposures seem to play a secondary role relative to intrinsic clinical determinants in populations characterised by advanced disease stages. However, in line with the exploratory nature of the analyses, the absence of an immediate relationship between short-term IAQ and severe exacerbations should not be interpreted as a lack of relevance of indoor exposures. Rather, it reflects the temporal divide between environmental exposure and clinical manifestation, as well as the multifactorial nature of exacerbation risk. In this context, environmental exposures can be conceptualised within the broader framework of cumulative and long-term influences on disease trajectory,^50^ although this study was not designed to formally assess such effects. In addition, the intrinsic characteristics of the study design may have limited the ability to detect measurable associations at this stage. These analyses were not designed to establish causal relationships but to provide preliminary, hypothesis-generating insights. By focusing exclusively on hospitalisations for severe exacerbations, the analysis intentionally captured only the most critical outcomes, thereby excluding a large proportion of mild and moderate events typically managed in community or home settings. Importantly, acute respiratory exacerbations are well known to be strongly driven by infectious triggers, which represent the predominant cause of acute deterioration in this population, and by oxidant pollutants (eg, NO₂ and O₃), which have been consistently associated with increased respiratory events and may act synergistically with PM and other airborne compounds to amplify airway inflammation.^51^

#### Towards hybrid care: digital and clinical strategies for prevention

Preventing hospitalisations in patients with advanced chronic respiratory disease may benefit from a shift towards early detection and proactive management of community-based exacerbations. This approach is being explored within the ongoing hybrid care intervention implemented in the study cohort,^28^ that combines remote and in-person care, coordinated by a nurse case manager and enabled by a digital adaptive case management platform.^52^ In daily clinical practice, this structure has the potential to enhance the early recognition and management of acute episodes in the community, thereby reducing hospital dependence and enabling more continuous, patient-centred follow-up. However, these capabilities are still under clinical evaluation. Preliminary experience suggests that integrating routine assessments with remotely collected data on lung function, symptom trajectories and heart rate variability (HRV) could contribute to a more objective basis for identifying early signs of deterioration. The evolving profiles of these parameters from baseline to recovery may, in the future, support data-driven stratification of exacerbation risk and inform personalised, anticipatory management within hybrid care models.

### Strengths, limitations and future research

This study represents one of the first large-scale deployments of LCS for household IAQ monitoring in patients with advanced chronic respiratory disease, integrated within a digitally enabled hybrid care model. The results provide early but solid evidence on the feasibility, robustness and clinical applicability of remote IAQ assessment, offering practical insights that can guide both research and clinical adoption.

Several limitations should be acknowledged. First, the temporal misalignment between short-term IAQ monitoring and retrospective clinical outcomes constrains causal interpretation and may have influenced the ability to detect associations. Second, exposure assessment was based on a limited monitoring period, which may not fully reflect longer-term exposure patterns or seasonal variability, particularly for pollutants such as formaldehyde, whose concentrations are influenced by temperature-dependent off-gassing dynamics. Third, although sensor performance was validated, some degree of measurement error and exposure misclassification cannot be excluded, especially in cases near category thresholds.

Building on these findings, the ongoing work will expand towards a prospective, longitudinal analysis that integrates environmental and clinical dimensions across the full follow-up period, incorporates broader health outcomes and community-level impact indicators, and refines exacerbation evaluation through patient-reported outcomes and physiological metrics such as oscillometry and HRV. Parallel efforts will focus on characterising dwelling structures, household behaviours and pollution sources following IPCHEM guidelines, as well as integrating contextual exposure determinants, including geolocation-based OAQ estimates, neighbourhood-level environmental indicators and socioeconomic variables where available. This will allow future analyses to better distinguish indoor source-driven pollution from outdoor infiltration, account for relevant social and environmental confounding and evaluate the combined contribution of indoor, outdoor and contextual exposures to respiratory outcomes. In addition, future developments will aim to translate monitoring into action through targeted patient-centred initiatives, including educational and tobacco-cessation campaigns, real-time feedback tools to promote ventilation awareness and a pilot household air-filtration protocol in highly polluted homes. Together, these next steps aim to consolidate the clinical value of IAQ monitoring and support its integration into future preventive, digitally enabled respiratory care.

## Conclusions

This study demonstrates the feasibility and operational applicability of LCS for short-term household IAQ monitoring in patients with chronic respiratory disease. Indoor air pollution was found to be highly prevalent, predominantly driven by fine PM and indoor smoking, highlighting the presence of modifiable exposure sources in this population.

While no associations were observed between IAQ metrics and severe clinical outcomes, these analyses should be interpreted as exploratory, given the study design. Further research is needed to evaluate the relationship between indoor environmental exposures and clinical outcomes using longitudinal and temporally aligned approaches.

Overall, these findings support the potential role of IAQ monitoring as a tool for exposure assessment and preliminary risk identification, while its integration into preventive or personalised care requires further investigation.

## Supplementary material

10.1136/bmjresp-2025-003899 online supplemental file 1

## Data Availability

Data are available on reasonable request.
